# Epidemiology of self-medication in Ethiopia: a systematic review and meta-analysis of observational studies

**DOI:** 10.1186/s40360-018-0248-8

**Published:** 2018-09-10

**Authors:** Mekonnen Sisay, Getnet Mengistu, Dumessa Edessa

**Affiliations:** 1 Department of Pharmacology and Toxicology, School of Pharmacy, College of Health and Medical Sciences, Haramaya University, P.O. Box 235, Harar, Ethiopia; 2 Pharmacology Unit, Department of Pharmacy, College of Medicine and Health Sciences, Wollo University, P.O. Box 1145, Dessie, Ethiopia; 3 Department of Pharmacy Practice, School of Pharmacy, College of Health and Medical Sciences, Haramaya University, P.O. Box 235, Harar, Ethiopia

**Keywords:** Self-medication, Epidemiology, Ethiopia

## Abstract

**Background:**

Self-medication is the use of drugs to treat self-diagnosed disorders and/or symptoms, or the intermittent or continued use of a prescribed drug for recurrent disease or symptoms. This phenomenon is alarmingly increasing over time despite the occurrence of health-related hazards. This study is, therefore, aimed to quantitatively estimate self-medication practice and possible reasons for it in Ethiopia.

**Methods:**

Data were identified from major databases and indexing services including EMBASE (Ovid), PubMed, MEDLINE (Ovid), and Google Scholar. Both published and unpublished records addressing self medication practice in Ethiopia without time limit were included for the study. Data were extracted with structured format prepared in Microsoft Excel and exported to OpenMeta[analyst] version 3.3 software for analyses. Pooled estimation of outcomes was performed with DerSimonian-Laird random-effects model at 95% confidence level. Sensitivity and subgroup analyses were also considered. Degree of heterogeneity of studies was presented with I^2^ statistics. Publication bias was also performed with the help of Comprehensive Meta-Analysis version-3 software and presented with funnel plots of standard error supplemented by Begg’s and Egger’s tests. The study protocol is registered on PROSPERO with reference number ID: CRD42018093790.

**Results:**

A total of 27 studies with 9586 participants were included for the study. The pooled prevalence of self-medication in Ethiopia was found to be 44.0% (95% confidence interval [CI]: 35.1, 52.8). Geographical-based subgroup analysis revealed that the highest prevalence was observed at the capital of Ethiopia, Addis Ababa, 62.8% (95% CI: 42.3, 83.2). Population based analysis indicated that healthcare professionals and students were the main practitioners of self-medication. Besides, the prevalence of self-medication practice in pregnant women is approximately 22.9% (95% CI: 9.8, 36). The most common reasons to practice self-medication were previous experience of clients and/or familiarity of treatments, 31.3% (95% CI: 21.5, 41.1) and perceived mildness of the illness, 31.1% (95% CI: 26.0, 36.2). The pooled prevalence of analgesics, antimicrobial agents and gastrointestinal drugs were 46.1% (95% CI: 36.2, 56.1), 28.2% (95% CI: 19.6, 36.8), and 14.9% (95% CI: 7.8, 21.9), respectively.

**Conclusion:**

Self-medication practice becomes a common phenomenon in Ethiopia. The use of prescription-only medications including antimicrobial agents without medical consult has become alarmingly high. This practice will come with potential health related hazards including emergence of antimicrobial resistance. Therefore, there must be a multitude of strategies for reversing the current worrying trends of self-medication.

**Electronic supplementary material:**

The online version of this article (10.1186/s40360-018-0248-8) contains supplementary material, which is available to authorized users.

## Background

Self-medication is an important element of self-care [1]. It is the use of medications for self-diagnosed disorders and/or symptoms regardless of the prescription status or without any medical consult. It includes the use of leftover medications from previously prescribed treatment regimen or drugs obtained from family, relatives or friends [2]. An attempt to treat self-recognized illness with intermittent or continued use of prescribed medications for recurrent disease or symptoms without consulting the prescriber is also considered as self-medication [3]. It is usually chosen by consumers for symptoms that they consider as troublesome, but not sufficiently serious to justify medical consultation [3–5].

Although responsible self-medication helps reduce the cost of treatment and the total time to be spent in visiting health facilities as well as facilitate the emergency treatment of life threatening conditions, it will come with potential health-related hazards including delay in treatment, occurrence of adverse drug reactions, drug-drug interactions, and antimicrobial resistance (AMR), among others [6, 7]. From a public health perspective, it should also be stressed that non-responsible self-medication practices incur significant healthcare costs, particularly with the cost of adverse drug reactions and drug interactions indicating a real economic burden [6, 8]. World Health Organization (WHO) considers self-medication as a part of the self-care that helps efficient use of the troubled healthcare awareness system with all legal aspects taken into considerations [3, 5]. WHO points out that responsible self-medication requires the medicinal product to be supported with information describing how to take the medicine, possible side-effects, monitoring, possible interactions, warnings, duration of use, among others [1]. WHO also reports that purchase of prescription-only drugs without a prescription is far more common than the sale of over-the-counter (OTC) drugs and is a pervasive phenomenon in developing countries [9]. Generally, self-medication is considered as an inferior good at high-income levels and a normal-good at low income levels, and hence, shows a strong and robust negative health insurance effect [4, 9]. Even if there is a systematic review paper published on the area of self-medication practice in Ethiopia [10], it failed to quantitatively estimate (meta-analyze) the overall prevalence of primary and secondary outcome measures included in this study. Therefore, this study attempted to provide evidence-based findings on self-medication practice in Ethiopia.

## Methods

### Study protocol

The identification of records, screening of titles and abstracts as well as evaluation of eligibility of full texts for final inclusion was conducted in accordance with the Preferred Reporting Items for Systematic review and Meta-analysis (PRISMA) flow diagram [11]. PRISMA checklist [12] was also strictly followed while conducting this systematic review and meta-analysis. The completed checklist is provided as supplementary material (Additional file 1: Table S1). The study protocol is registered on PROSPERO with reference number ID: CRD42018093790; and the published methodology is available from: http://www.crd.york.ac.uk/PROSPERO/display_record.php?ID=CRD42018093790

### Data sources and search strategy

Literature search was carried out through visiting legitimate databases and indexing services-PubMed, MEDLINE (Ovid® interface), EMBASE (Ovid® interface) and other supplementary sources including Google Scholar, WorldCat catalog, ResearchGate and Cochrane library. Advanced search strategies were applied in major databases to retrieve relevant findings closely related to self-medication practice. Articles published in subscription based journals and indexed in Science-Direct and Wiley online library were accessed through HINARI interface. The search was conducted with the aid of carefully selected key-words and indexing terms without specification in time. Excluding the non-explanatory terms, the search strategy included “self-medication” [MeSH], “self remed*”, “self-treatment”, “self-care” “non prescription”, “over the counter”, OTC and “Ethiopia”. Boolean operators (AND, OR), and truncation were used appropriately for systematic identification of records for the research question. The search was conducted from 1 April –25 April, 2018 and all published and unpublished articles available online till the day of data collection were considered. Gray literatures from organizations and online university repositories were accessed through Google Scholar and WorldCat.

### Screening and eligibility of studies

Records identified from various electronic databases, indexing services and directories were exported to ENDNOTE reference software version 8.2 (Thomson Reuters, Stamford, CT, USA) with compatible formats. Duplicate records were identified, recorded and removed with ENDNOTE. Some duplicates were addressed manually due to variation in reference styles across sources. Thereafter, two authors (MS and GM) independently screened the title and abstracts with predefined inclusion criteria. Two authors (MS and DE) also independently collected full tests and evaluated the eligibility of them for final inclusion. In each case, the third author played a critical role in solving discrepancies arose between two authors and in coming to a final consensus.

### Inclusion and exclusion criteria

During initial screening of titles and abstracts as well as evaluating full texts for eligibility, there were predefined inclusion-exclusion criteria to come up with the final included articles. Observational studies addressing self-medication practice and conducted in Ethiopia regardless of sociodemographic characteristics were included. Only English language literatures were considered without time limits. Studies that addressed knowledge and/or attitude but not practice of self-medication were excluded during initial screening. Articles with irretrievable full texts (after requesting full texts from the corresponding authors via email and/or ResearchGate), records with unrelated outcome measures, articles with missing or insufficient outcomes were also excluded.

### Data extraction

With the help of standardized data abstraction format prepared in Microsoft Excel (Additional file 2: Table S2), two authors (MS and DE) independently extracted important data related to study characteristics (region and study area, first author, year of publication, study design, population characteristics, and sample size) and outcome of interest (effect size data including prevalence of self-medication, reasons for self-medication and common drugs used by self-medicated clients. The event rate (proportion) was calculated out of 1 and standard error of Logit event rate was also added with the help of Comprehensive Meta-analysis (CMA) version-3 software.

### Quality assessment of studies

The quality of studies was evaluated according to Newcastle-Ottawa scale adapted for Cross-sectional studies [13] and graded out of 10 points (stars). For ease of assessment, the tool included important indicators categorized in to three major sections: 1) the first section assesses the methodological quality of each study and weighs a maximum of five stars; 2) the second section considers comparability of the study and takes 2 stars; and 3) the remaining section assess outcomes with related statistical analysis. This critical appraisal was conducted to assess the internal (systematic error) and external validity of studies and to reduce the risk of biases. The mean score of two authors were taken for final decision and studies with score greater than or equal to five were included.

### Outcome measurements

The primary outcome measure in this meta-analysis is the prevalence of self-medication in Ethiopia. It is aimed to assess the pooled estimates of self-medication practice in the country. This study has also two secondary outcome measures: the reasons to practice self-medication and common drugs used for self-medication in Ethiopia. The sample size was intentionally adjusted to response rates and number of illnesses in individual study to reduce bias in calculating the overall prevalence. In case of secondary outcomes, the denominator was adjusted to self-medicated patients to extract justified reasons (events) that urged them to practice self-medication. Similar reasons were combined from each study for overall estimates. For determining the prevalence of drugs used for self-medication, the total number of drugs utilized by self-medicated patients in each study was considered for analyses.

### Data processing and statistical analysis

The relevant data were extracted from included studies using format prepared in Microsoft Excel and exported to OpenMeta[Analyst] advanced software (http://www.cebm.brown.edu/openmeta) for analyses of pooled estimate of primary and secondary outcome measures, as well as subgroup analysis. Considering variation in true effect sizes across population (clinical heterogeneity), Der Simonian and Laird’s random effects model was applied for the analyses at 95% confidence level. Heterogeneity of studies was assessed using I^2^ statistics. CMA version-3 software (Biostat, Englewood, New Jersey, USA) was used for publication bias assessment. The presence of publication bias was evaluated by using the Begg and Rank correlation as well as Egger’s regression tests and presented with funnel plots of standard error and precision with Logit event rate [14, 15]. A statistical test with a *p*-value less than 0.05 (one tailed) was considered significant.

## Results

### Search results

**Fig. 1:**
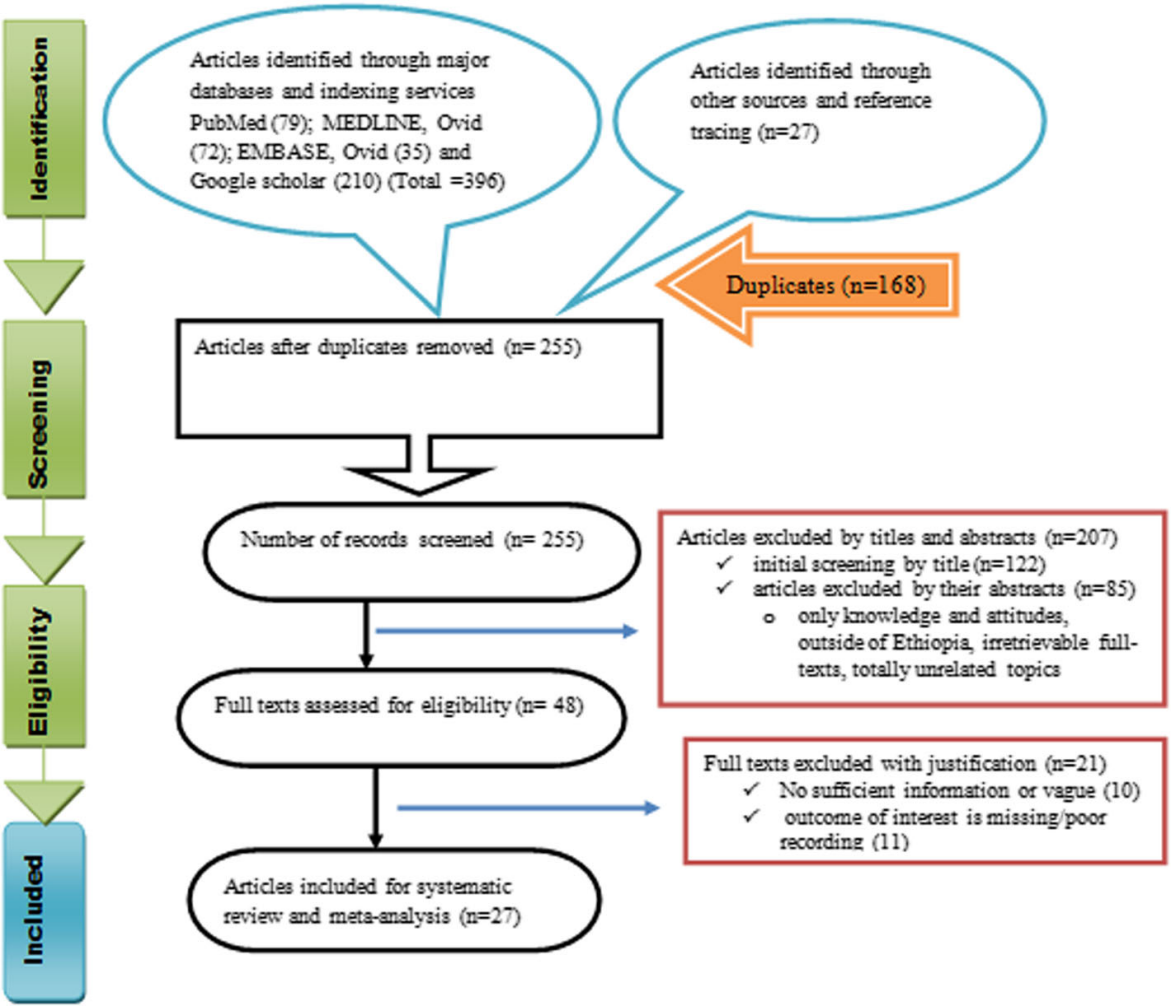
PRISMA flow chart depicting the selection process

A total of 423 records were identified from several sources. From these, 168 duplicate articles were removed with ENDNOTE. The remaining 255 records were screened using their titles and abstracts and 207 of them were excluded. Full texts of 48 records were then evaluated as per predetermined eligibility criteria for inclusion. Twenty one articles were also excluded as the outcome of interest was found missing, insufficient and/or ambiguous. Finally, 27 articles passed the eligibility criteria and quality assessment and hence included in the study (Fig. 1).

### Study characteristics

As shown in Table 1, a total of 27 studies with 9586 participants were included for systematic review and meta-analysis. From whom, 4105 clients practiced self-medication. All the included studies employed prospective cross-sectional study design in common. The year of publication of included studies ranges from 2001 to 2018. The study included a wide range of population characteristics. The adjusted sample size ranged from 136 in Butagira town [16] to 1190 participants in three towns of northwest Ethiopia [17]. The prevalence of self-medication ranged from 12.5% in Butagira [16] to 78.1% in Limmu Genet [18]. Regarding geographic distribution, the 27 studies were obtained from five regions and one city administration (Addis Ababa): nine studies were conducted in Oromia region [18–26], ten studies in Amhara [17, 27–35], three studies in Addis Ababa [36–38], two studies both in Tigray [39, 40] and Southern Nations Nationalities and Peoples of Ethiopia [16, 41] and one study from Harari region [42]. No study was included from Somali, Gambela, Benishangul-Gumuz and Afar regions as well as Dire Dawa city administration. Only one unpublished study was found to fulfill the inclusion criteria [35]. The average quality scores of studies ranges from 5 to 8.5 as per the Newcastle-Ottawa scale (Table 2).

**Table 1:** Characteristics of studies included for systematic review and Meta analysis

Region	Study area	Author	Publication year	Study design	Study Population	Sample size	Event	Prev (%)	Event rate	SE
Oromia	Nekemte town	Sado et al. [19]	2017	CS	health professionals	154	104	67.5	0.675	0.17
	Sire town	Jaleta et al. [20]	2016	CS	urban dwellers	243	66	27.16	0.272	0.14
	Jimma University	Angamo and Wabe [21]	2011	CS	Medical science students	403	95	23.6	0.236	0.12
	JUSH	Befkadu et al [22]	2014	CS	Pregnant women	303	61	20.1	0.201	0.14
	Assendabo town	Suleman et al [23]	2009	CS	Urban residents	143	56	39	0.390	0.17
	Arsi University	Bekele et al. [24]	2016	CS	Health science students	388	299	77.1	0.771	0.12
	Jimma town	Worku & G/mariam [25]	2003	CS	Urban residents	152	42	27.6	0.276	0.18
	Jimma town	Ararsa ad Bekele [26]	2015	CS	Private Pharmacy clients	312	242	77.57	0.776	0.14
	Limmu Genet	Bekele et al. [18]	2018	CS	Urban residents	304	237	78.1	0.781	0.14
Amhara	University of Gondar	Abay and Amelo [27]	2010	CS	Medical and health Science students	213	82	38.5	0.385	0.14
	University of Gondar	Gelaye [28]	2017	CS	Social science students	385	126	32.7	0.327	.11
	Debre Markos University	Dilie et al. [29]	2017	CS	Health science students	250	146	58.4	0.584	0.13
	Kolladiba town	Abrha et al. [30]	2014	CS	Heads of households	261	164	62.8	0.628	0.13
	Dessie town	Baye and Sada [31]	2018	CS	Urban dwellers	370	157	42.4	0.424	0.11
	Gondar, Kolladiba and Debark	Abula and Worku [17]	2001	CS	Urban dwellers	1190	324	27.22	0.272	0.07
	Bahirdar	Abeje et al. [32]	2015	CS	Pregnant mothers	356	128	36	0.360	0.11
	Bahirdar	Gebeyehu [33]	2015	CS	Urban residents	388	70	18	0.180	0.13
	Bahirdar	Mihrete [35]		CS	Urban residents	595	76	12.8	0.128	0.12
	Meket (North Wollo)	Kassie et al. [34]	2018	CS	Inhabitants of the district	722	259	35.9	0.359	0.08
SNNPR	Butagira	Gedif and Hahn [16]	2003	CS	Mothers	136	17	12.5	0.125	0.26
	Werabe	Mossa et al. [41]	2012	CS	Heads of house holds	225	78	34.6	0.346	0.14
Tigray	Mekele University	Eticha [39]	2014	CS	Adi-haqi campus students	407	181	44.5	0.445	0.10
	Mekele University	Gutema et al. [40]	2011	CS	Health science students	148	64	43.24	0.432	0.17
Addis Ababa	Addis Ababa	Shafie et al. [37]	2018	CS	Urban residents	604	456	75.5	0.755	0.09
	Addis Ababa	Gedif and Hahn [36]	2002	CS	Heads of households	254	94	37	0.370	0.13
	Rift Valley University	Beyene et al. [38]	2017	CS	Pharmacy students	443	334	75.39	0.754	0.11
Harari	Harar Health Science College	Hailemichael et al. [42]	2016	CS	Health science students	237	147	62	0.620	0.13
Total						9586	4105			

*CS* cross-sectional, *Prev* prevalence, *SE* standard error, *JUSH* Jimma University Specialized Hospital, *SNNPR* Southern nations, nationalities and peoples region

**Table 2:** Quality assessment of included studies using Newcastle-Ottawa scale adapted for cross-sectional studies

Study ID	Methodological quality (5)	Comparability (2)	Outcomes measures and analysis (3)	Total (10)
Abay and Amelo	3	1	2	6
Abeje et al	4	1	2.5	7.5
Abrha et al.	2.5	1	2	5.5
Abula and Worku	2.5	1	1.5	5
Angamo and Wabe	3	1	2	6
Ararsa ad Bekele	3	1	2	6
Baye and Sada	3.5	1	1.5	6
Befkadu et al	2	1	2	5
Bekele et al., 2016	4	1	2	7
Bekele et al., 2018	4	1	3	8
Beyene et al.	2	1	2	5
Dilie et al.	4	1	1.5	6.5
Eticha et al	4	1	1.5	6.5
Gebeyehu et al	4	1	2.5	7.5
Gedif and Hahn, 2002	3	1	1.5	5.5
Gedif and Hahn, 2003	3.5	1	2	6.5
Gelayee	4	1	2.5	7.5
Gutema et al.	2.5	1	1.5	5
Hailemichael et al.	4	1	1.5	6.5
Jaleta et al.	3	1	2	6
Kassie et al	4.5	1.5	2.5	8.5
Mihrete	3	1	1	5
Mossa et al.	2.5	1	1.5	5
Sado et al.	4	1	3	8
Shafie et al.	4.5	1	3	8.5
Suleman et al.	3	1.5	2	6.5
Worku and G/mariam	2.5	1	2	5.5

*NB* the numbers in parenthesis are maximum scores to be given per category

### Study outcome measures

#### Primary outcomes

**Fig. 2:**
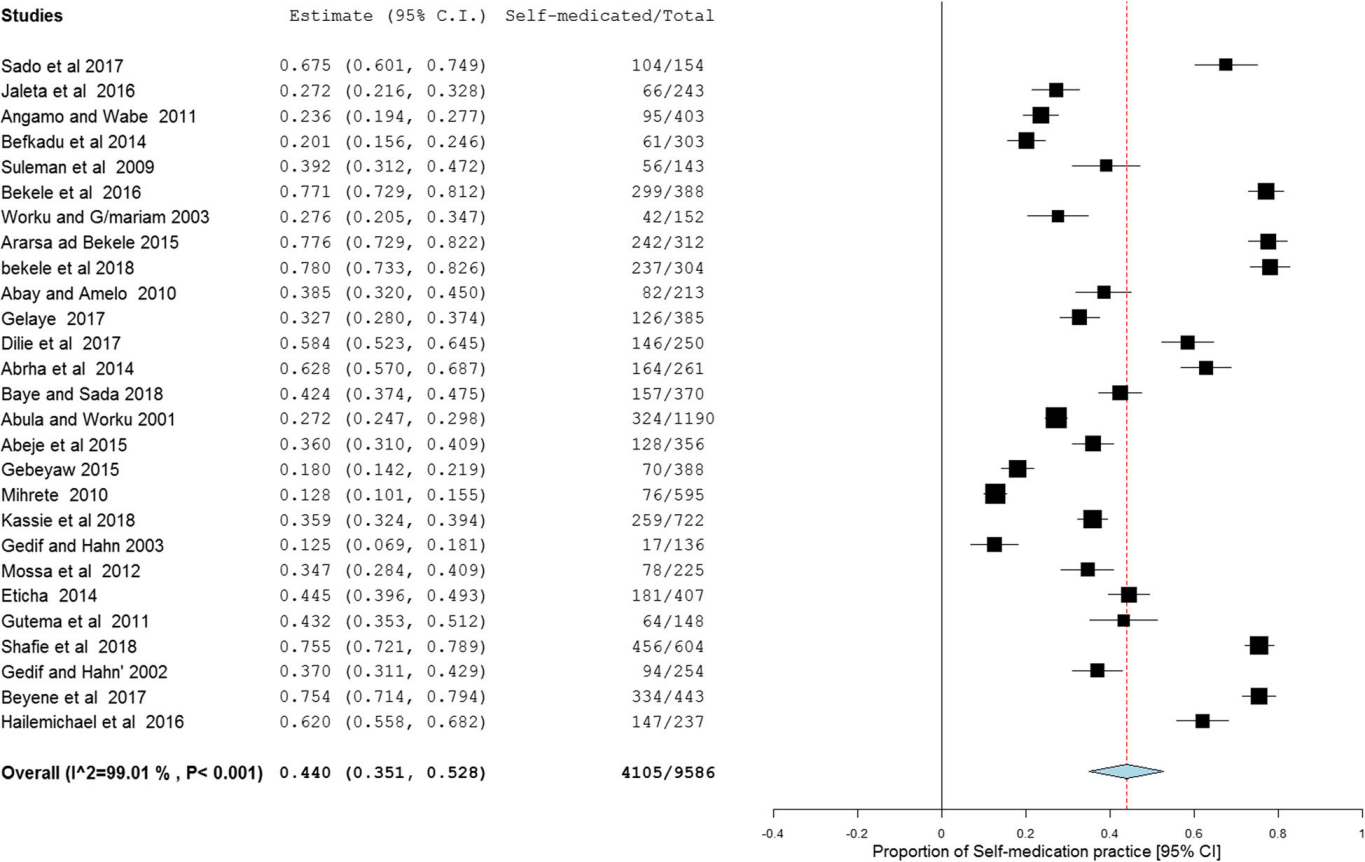
Forest plot illustrating the pooled analysis of 27 studies reporting self medication practice in Ethiopia

From the 27 studies describing self-medication practice, the pooled prevalence of self-medication in Ethiopia was found to be 44.0% (95% confidence interval [CI]: 35.1, 52.8%). As the I^2^ statistic revealed, there is a high degree of heterogeneity across studies (I^2^ = 99.01%, *p* < 0.001). Random effects model was assumed for this meta-analysis (Fig. 2).

#### Sensitivity and subgroup analyses

**Fig. 3:**
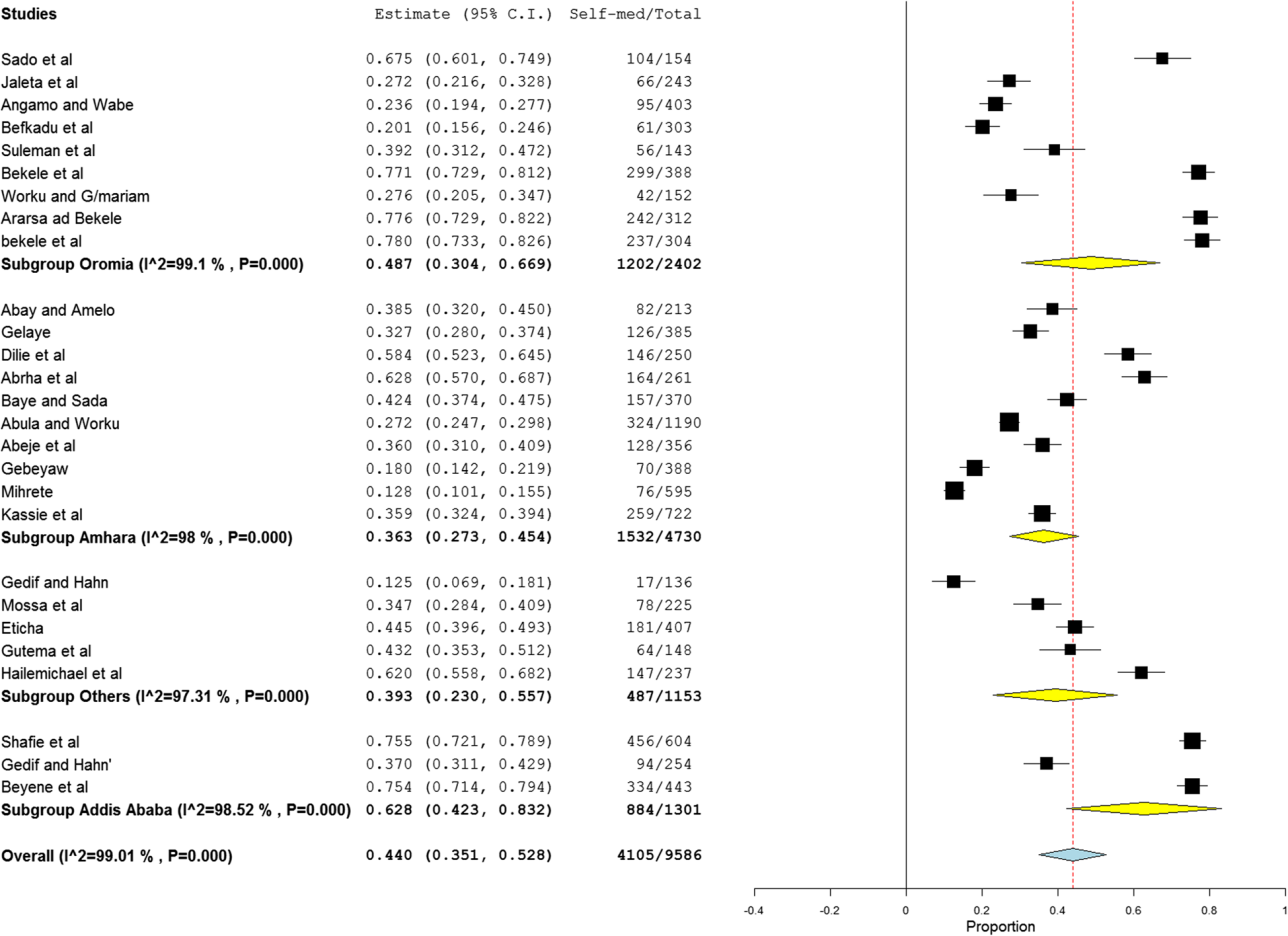
Subgroup analysis of studies describing the prevalence of self medication segregated by geographical distribution. Others include studies conducted in Tigray, Harari and Southern Ethiopia

**Fig. 4:**
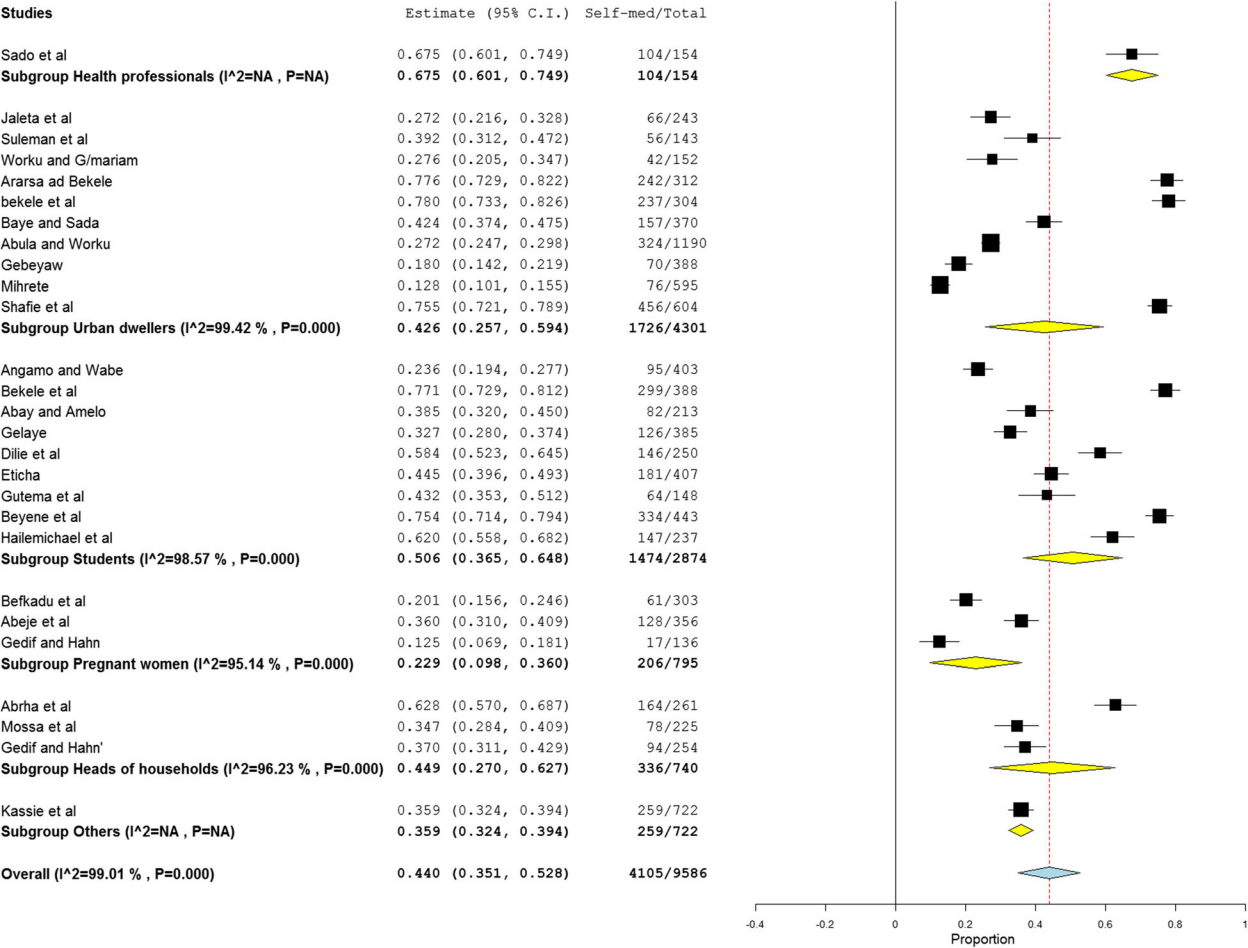
Subgroup analysis of self medication by population characteristics. Other, inhabitants of Meket district

**Fig. 5:**
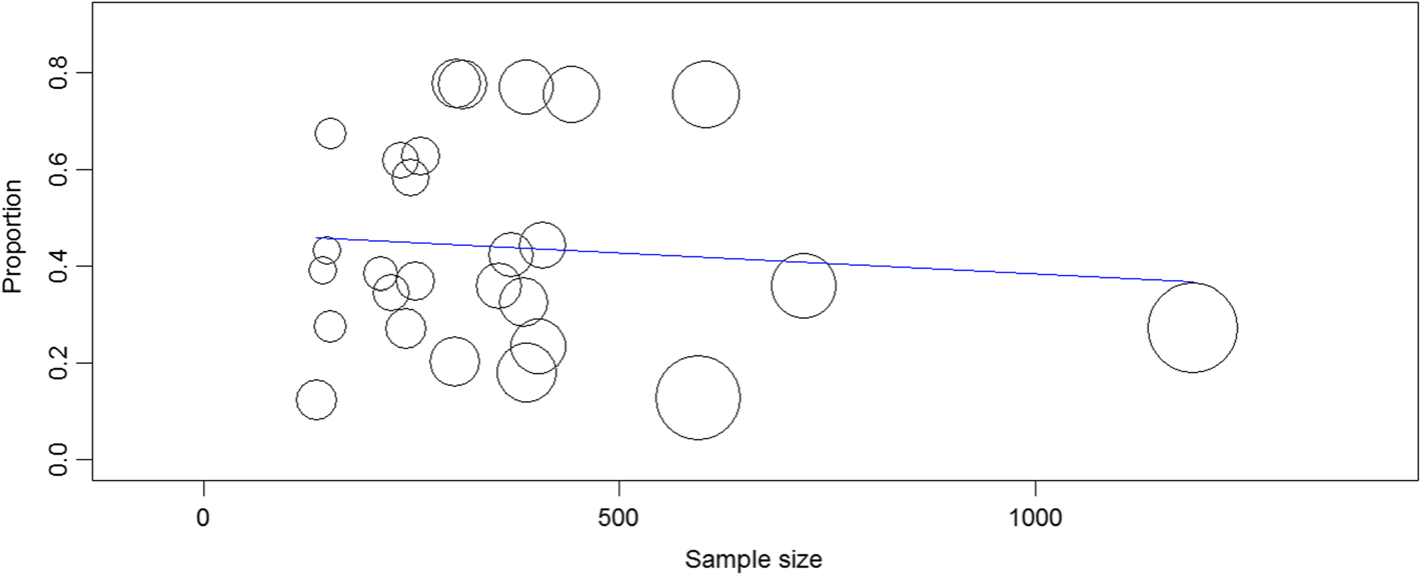
Univariate meta-regression model using sample size

There was no any significant change on the degree of heterogeneity even if we attempted to exclude the expected outliers as well as one or more of the studies from analysis. Therefore, we are subjected to include all the studies for the meta-analysis. We also conducted a subgroup analysis based on geographical distribution and population characteristics. Subgroup analysis based on region revealed that the highest self-medication prevalence was observed at the capital of Ethiopia, Addis Ababa, 62.8% (95% CI: 42.3, 83.2) followed by Oromia region with prevalence of 48.7% (95% CI: 30.4–66.9). Relatively, a lower pooled estimate was observed in Amhara region 36.3% (95% CI: 27.3–45.4) as depicted in the forest plot (Fig. 3). Another subgroup analysis with population characteristics showed that health professionals and students have been the major users of self-medication followed by urban dwellers. The study revealed that the prevalence of self-medication practice in pregnant women is approximately 22.9% (95% CI: 9.8, 36) which seems relatively lower compared to other groups but clinically concerning result in Ethiopia (Fig. 4). Time-based subgroup analysis indicated that there is an alarmingly increasing practice for the last two decades: 28.5% (95% CI: 20.4, 36.5) in before 2010, 34.9% (95% CI: 23.0, 46.8) within 2010–2014 and 54.5% (95% CI: 42.4, 66.7) from 2015 to April 2018 (Table 3). Univariate meta-regression revealed that sampling distribution is not a source of heterogeneity (regression coefficient = 0.000, *p*-value = 0.634) (Fig. 5).

**Table 3:** Subgroup analysis based on the publication year of studies

Year of publications	Number of publications	Pooled estimate (95% CI)	Heterogeneity (I^2^)
Before 2010	5	28.5% (20.4, 36.5)	99.0%
2010–2014	8	34.9% (23.0, 46.8)	97.8%
2015–2018	14	54.5% (42.4, 66.7)	98.9%
Overall	27	44.0% (35.1, 52.8)	99.01%

*CI* confidence interval

#### Secondary outcomes

Following the pooled estimate of self-medication practice, we conducted secondary analyses for the possible reasons that necessitate for practicing self-medication and most common drug classes being used by such clients. We tried to combine similar/related reasons from various studies with the number of self-medicated clients. The number of events (reasons) and total self-medicated clients were considered for data entry. The most common reasons justified for self-medication in Ethiopia were previous experience of clients and/or familiarity of treatments, 31.3% (95% CI: 21.5, 41.1) and perceived mildness of the illness that did not push them to visit health facilities, 31.1% (95% CI: 26.0, 36.2). The remaining reasons were affordability of self-medication, 26.6% (95% CI: 17.0, 36.1), saving of time, 19.4% (95% CI: 14.1, 24.7), suitability for emergency treatment, 16.6% (95% CI: 9.4, 23.8) and less expectation to modern health facilities, 13.6% (95% CI: 8.3, 18.9) (Table 4). The study also addressed common classes of medication being used for self-treatment without authorized prescription and regardless of status of prescription. The pooled prevalence of analgesics and anti-inflammatory drugs, antimicrobial agents and gastrointestinal drugs were 46.1% (95% CI: 36.2, 56.1), 28.2% (95% CI: 19.6, 36.8), and 14.9% (95% CI: 7.8, 21.9), respectively (Table 4).

**Table 4:** Pooled estimates of secondary outcome measures among self-medicated clients

Secondary outcome measures		Pooled estimate (95% CI)	Heterogeneity (I^2^)
Justified reasons for practicing self-medication	Previous experience or familiarity of treatments	31.3% (21.5, 41.1)	97.95%,
	Perceived mildness of the illness	31.1% (26.0, 36.2)	85.41%
	Affordability of self-medication	26.6% (17.0, 36.1)	98.02%
	Time saving nature	19.4% (14.1, 24.7)	91.27%
	Suitability for emergency care	16.6% (9.4, 23.8)	93.83%
	Less expectation in health facilities	13.6% (8.3, 18.9)	73.59%
Top three classes of drugs used	Analgesic and anti-inflammatory drugs	46.1% (36.2, 56.1)	95.91%
	Antimicrobial agents	28.2% (19.6, 36.8)	96.56%
	Gastrointestinal agents	14.9% (7.8, 21.9)	95.06%

*CI* confidence interval

#### Publication bias

**Fig. 6:**
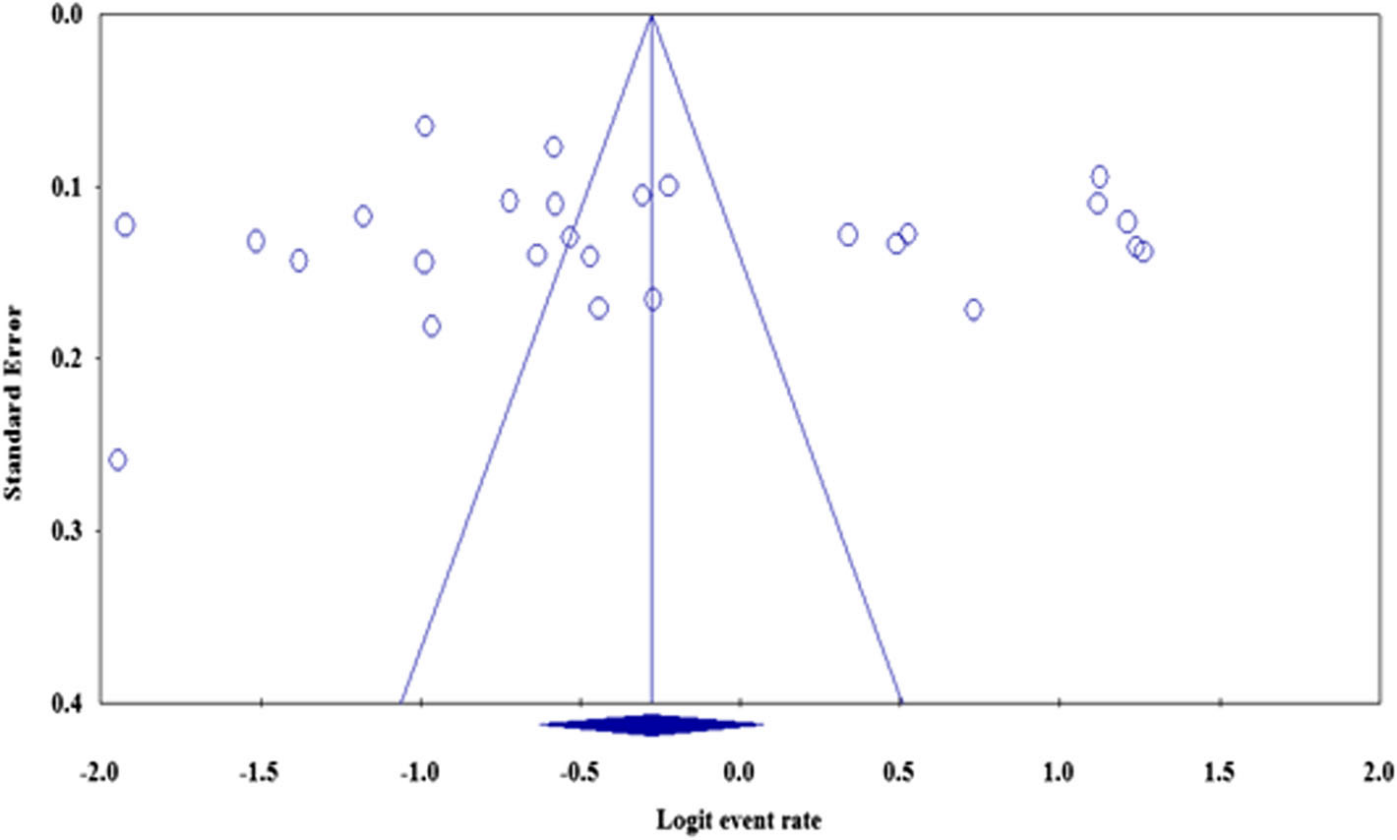
Publication bias using funnel plot of standard error by Logit event rate

Funnel plots of standard error with logit effect size (event rate in this case) supplemented by statistical tests confirmed that there is no evidence of publication bias on studies reporting self-medication practice in Ethiopia (Egger’s regression test (one-tailed), *p* = 0.362; Begg’s correlation test (one tailed), *p* = 0.353) (Fig. 6).

## Discussion

Regardless of sociodemographic characteristics of the study participants, we attempted to analyze 27 original studies addressing self-medication practice in Ethiopia. From this, 9586 participants were included for pooled estimation of primary outcome measures. Since self-medication is a common phenomenon in low-income countries like Ethiopia where resources are often scarce, we tried to include all eligible studies for better power of estimation and ease of subgroup analyses. The overall prevalence of self-medication in Ethiopia was found to be 44.0% (95% CI: 35.1, 52.8). This finding indicated that more than two patients out of five practiced self-medication. Self-medication practice is also alarmingly increasing over time. Comparing the prevalence of the last two decades, the practice has been doubled from 28.5% (before 2010) to 54.5% (2015 to April 2018).

Responsible self-medication is an important response to medical condition which should be controlled in its early stage. However, the irrational use of medications may lead to devastating consequences including toxicity, drug interactions and AMR [43]. Approximately, 26.7% adverse drug reactions were reported among elderly who practiced self-medication in Mexico [44]. In low-income countries, most diseases are being treated by self-medication. It is preceded with inadequate or lack of clinical evaluation of patients, which could result in misdiagnosis and delays in appropriate treatment. This will again incur patients to several direct and indirect healthcare-related costs [3–5]. The temporal increment of self-medication practice might be related to the advancement of information technology and high access of drugs from several sources which make the patients to be closer to disease and drug related information. Generally, drugs can be obtained from families, friends, neighbors, dispensers, previously prescriptions (leftover drugs), or suggestions from an advertisement and promotional activities [6, 7]. Since, this is the first meta-analysis (quantitative synthesis) regarding epidemiology of self-medication in Ethiopia; we tried to compare our finding to other national surveys and reviews conducted outside of Ethiopia. The practice is far more than twice the finding reported from Brazil where the overall national prevalence was 18.3% [45].

Population-based subgroup analysis indicated that healthcare professionals as well as university and college students have been the main users of self-medication. To this end, self-medication was found to be a significant concern for both healthcare practitioners and medical students [46]. A systematic review of 27 studies addressing self-medication practice in students in Greece, reported that self-medication was approximately 50%. Despite sociodemographic indices, this result is in trajectory with our finding (50.6%) [47]. National wide surveys in Turkey and Serbia also indicated that the prevalence of self-medication among university students were 63.4 and 79.9%, respectively and the later being among medical students [46, 48]. Generally, the prevalence of self-medication is alarmingly high among students of health-related disciplines, despite knowing the consequences and potential risks [49]. It becomes evident that self-medication is strongly embedded within the culture of both healthcare professionals (physicians in particular) and medical or health science students. Inappropriate self-medication represents a serious threat to professionalism within medicine and health sciences and has the potential to erode the public’s trust in the profession. This study also revealed that one in five pregnant women practiced self-medication without medical consultation. This will come with serious health related risks for such special population. It should be stressed that drug use during pregnancy must consider the potential benefit (s) to the mother and the potential risk (s) to the embryo or fetus and must be assisted with critical medical advice [50].

Coming to secondary outcome measures, the pooled estimates revealed that the top six reasons provided by self-medicated clients in descending order were previous exposure/familiarity with the treatments (31.3%), perceived mildness of illnesses (31.1%), affordability of self-medication (26.6%), time saving nature (19.4%), suitability to emergency-care (16.6%) and less expectation to health facilities (13.6%). In concordant with this study, study done in Brazil indicated that previous experience and/or familiarity with medication was the leading reason for self-medication [45]. Similarly, the reasons for self-medication among healthcare professionals were easy availability of drugs, professional exposure to drugs and knowledge of treatment of the diseases [49]. Income level was also a major determinant of self-medication [51]. It becomes evident that the use of OTC drugs in low-income countries is more frequent due to accessibility, cultural customs and a perceived saving of time and money compared to consulting a healthcare professionals [1, 52].

As per the pooled point estimates, top three classes of drugs used by self-medication practitioners were analgesics and anti-inflammatory agents (46.1%), antimicrobials (28.2%) and gastrointestinal drugs (14.9%). In line with this finding, non-steroidal anti-inflammatory drugs and analgesics were the top self-medicated drugs [49, 53] for conditions such as fever, chills, headache and common cold. Even if majority of these drugs are regarded as OTC (non-prescription), appropriate consultation is mandatory from prescriber and/or dispenser to prevent potential risks of self-medication. Moreover, the use of prescription-only medications including antimicrobials becomes a prevailing practice in Ethiopia. WHO reports that self-medication with antimicrobial agents is becoming widespread, with one of its greatest risk being AMR [52]. Self-medication with antimicrobials has been found a common practice in low-income countries and it has been shown to be significantly related to many factors including availability and accessibility, lack of access to health care facilities, high prevalence of infectious diseases poor awareness, poor regulation, and lack of supervision by health professionals [54]. Perceptions, knowledge and attitudes about AMR in self-medication practitioners might help the policy-makers and regulatory authorities to develop educational programs directed to change the perceptions and attitudes about the appropriate use of antimicrobials [55, 56].

## Conclusion

Self-medication becomes a common phenomenon in Ethiopia. The use of prescription-only medications including antimicrobial agents without medical consult has become alarmingly high. This practice will come with potential health related hazards including emergence of AMR. Besides, temporal increment and high prevalence of self-medication among health professionals and students is also a major bottleneck for Ethiopia. This complex self-medication behavior could be regarded as an occupational hazard for the medical profession. Even though, the positive aspects of self-medication related to primary healthcare should be recognized in low-income countries, the risk may outweigh the benefit if the current trend continues. In Ethiopia, as self-medication is treated as a normal good, expanding coverage of public health insurance to a broader group of citizens and at the same time imposing some form of regulatory measures should slow down the use of self-medication. In addition, proper and adequate training of pharmacy staffs to diagnose, treat, and refer patients would be crucial in community drug retail outlets. These and other related strategies should be designed to reverse the current worrying trends of self-medication.

## Additional files

Additional file 1: **Table S1.** Completed PRISMA checklist. The checklist highlights the important components addressed while conducting systematic review and meta-analysis from observational studies. (DOC 65 kb)

Additional file 2: **Table S2.** Data abstraction format. The table presented the ways of data collection (study characteristics and outcome measures) in Microsoft excel format. It also contained a raw data for primary and secondary outcomes analyses. (XLSX 24 kb)

## Abbreviations

AMR: Antimicrobial resistance
CMA: Comprehensive meta-analysis
MeSH: Medical subject headings
OTC: Over the counter
PRISMA: Preferred Reporting Items for Systematic reviews and Meta-Analysis
WHO: World Health Organization
